# Essential cancer medicines and cancer outcomes: Cross‐sectional study of 124 countries

**DOI:** 10.1002/cam4.6642

**Published:** 2023-10-30

**Authors:** Oghenefejiro (Theresa) Ikpeni, Darshanand Maraj, Hannah Woods, Aine Workentin, Christopher M. Booth, Nav Persaud

**Affiliations:** ^1^ MAP Centre for Urban Health Solutions St. Michael's Hospital Toronto Ontario Canada; ^2^ Department of Oncology Queen's University Kingston Ontario Canada; ^3^ Department of Family and Community Medicine University of Toronto Toronto Ontario Canada

## Abstract

**Background:**

Cancer is the second leading cause of death worldwide. Alongside other interventions, access to certain medicines may decrease cancer‐associated mortality. Listing medicines on national essential medicines lists may improve health outcomes. We examine the association between cancer mortality amenable to care and the listing of cancer medicines on national essential medicines lists (NEMLs) of 124 countries.

**Methods:**

In this cross‐sectional study, we determined the number of medicines used to treat eight cancers on NEMLs and used multiple linear regression to analyze the association between cancer health outcome scores and the number of medicines on NEMLs while controlling for GDP. A sensitivity analysis was also conducted using selected medicines.

**Findings:**

The number of cancer medicines on NEMLs was not associated with cancer health outcome scores when GDP was controlled for non‐melanoma skin (*p* = 0.224), uterine (*p* = 0.221), breast (*p* = 0.145), Hodgkin's lymphoma (*p* = 0.697), colon (*p* = 0.299), leukemia (*p* = 0.103), cervical (*p* = 0.834), and testicular cancers (*p* = 0.178).

**Interpretation:**

There was a weak association between listing medicines for eight cancers in NEMLs and amenable mortality. Further studies are required to explore association between cancer health outcomes and other factors such as actual availability of medicines listed, access to surgeries, accurate diagnosis, radiotherapy, and early detection.

## 1 INTRODUCTION

Cancer is the second cause of death globally and accounts for 17% of worldwide deaths.^1^ The global cancer burden is estimated to have risen to 19.3 million new cases and 10.0 million deaths in 2020.^1^ One in 5 people worldwide develop cancer during their lifetime, and one in 8 men and one in 11 women die from the disease.^2^ Highly effective medicines are one part of care that prevents deaths arising from a number of cancers.^3^ The World Health Organization (WHO) therefore includes essential medicines for cancer treatments on its Model List of Essential Medicines (WHO EML).^4^ Essential medicines are those that satisfy the priority health care needs of the population and are “selected with due regard to disease prevalence and public health relevance, evidence of clinical efficacy and safety, and comparative costs and cost‐effectiveness.”^5^ In 1977, the WHO EML initially listed seven anticancer medicines; this number has increased over time and presently it lists 62 medicines used in the management of cancers.^6^ For the management of cancer, the WHO EML lists alkylating agents, antitumor antibiotics, antimetabolites, mitotic inhibitors, anthracyclines, hormones and antagonists, other cytotoxic drugs, biological agents, and supportive therapy (such as medicines used in the management of pain associated with cancers).^4^ The WHO recommends countries to establish national essential medicines lists (NEMLs) as they select medicines intended to be “available within the context of functioning health systems at all times in adequate amounts, in the appropriate dosage forms, with assured quality, and at a price the individual and the community can afford.”^5^ These lists influence access to selected medicines that meet the priority health care needs of countries as they are often the primary resource used for selection of medicines in the public sector where many patients are managed for cancers.^7^

Access to quality health care has shown to improve several health outcomes.^8^, ^9^ Health care and access quality can be estimated using amenable mortality, which measures mortality rates from causes that should not be catastrophic in the presence of adequate health care.^10^, ^11^, ^12^, ^13^ Amenable causes include communicable diseases (e.g., tuberculosis, upper respiratory tract infections) and non‐communicable diseases (NCDs) (e.g., cancers).^3^ Amenable mortality can be therefore used as a tool to assess the performance of health care systems worldwide.^14^

The purpose of this study was to determine if there is an association between listing medicines used in the management of 8 cancers and amenable mortality for these conditions measured by the health care access and quality (HAQ) score.^3^

## 2 METHODS

### 2.1 Dataset sources

NEMLs from the WHO's National Essential Medicines Lists Repository were obtained and all medicines listed for each country, with some exceptions, were recorded. These lists were used to create a Global Essential Medicines (GEM) database of 2182 medicines.^15^, ^16^

Each country was assigned a HAQ health outcomes score for each of the eight cancers.^3^ The HAQ score is based on the Global Burden of Diseases Study, and the disease specific HAQ scores for cancers are based on mortality‐to‐incidence ratios that utilize cancer registry data for each country.^3^ Each HAQ score is age‐standardized and scaled to range from 0 to 100 based on the percentile in comparison with other countries.

### 2.2 Data collection

To create a list of medications used in the management of eight common cancers that are substantial contributors to global mortality (breast, non‐melanoma [NM] skin, colon, Hodgkin's, Leukemia, testicular, uterine, and cervical cancers), we searched the WHO website for international treatment guidelines for the management of cancers in June 2019. Three guidelines published by the WHO were used to provide guidelines for the management of NCDs and one for breast cancer. The guidelines selected were WHO Package of Essential Non communicable (PEN) Diseases Interventions for Primary Health Care in Low‐Resource Settings,^17^ “Best Buys” and other recommended interventions for the prevention and control of non‐communicable diseases^18^ and WHO EMRO Technical Publication series 31‐Guidelines for management of breast cancer.^19^ For the other seven cancer causes which did not have an international treatment guideline, medicines retrieved from MIA and MEDI‐HPS searches based on ICD‐9/10 codes mapped to causes were included if determined to be clinically appropriate to treat the conditions associated with the cause. If more than 2 medications from a drug class (based on ATC codes) appeared in the searches for a cause, all other drugs from that class were included in the final list if determined to be clinically appropriate as well. The 20th WHO EML^4^ along with these guidelines were used to create a final list of medications for each cancer cause after duplicates were collapsed.

### 2.3 Data extraction

The list of medications was reviewed by a medical oncologist (CB) to ensure they were correctly matched to specific cancers. The list of the medications associated with each of the eight cancers were coded into the GEM database^15^ and medications overlapping on these lists and each country's NEML were identified, totaled, and a coverage score for each country per cancer cause was created. Data for gross domestic product (GDP) per capita was obtained from the World Bank for the year 2016. World bank data from 2016 was used to match the 2017 NEML as it was the closest year to 2017 with available data.^20^

### 2.4 Data analysis

Statistical analysis was carried out in R version 3.6.0, and for this model, the co‐efficient for each medicine and covariate, the lower 95% CI, the upper 95% CI, and *p*‐value of the association were recorded. For this analysis, GDP per capita in international dollars was included as a covariate. GDP represents health expenditure and is generally known to influence health systems. Multiple linear regression was used to assess the association between the HAQ cancer health outcome scores and the number of medicines listed in each country's NEML for each cancer cause. Linear regression was run both unadjusted and adjusted with GDP as a covariate. Countries were excluded from the analysis if data for the cancer health outcome and covariate were unavailable.

We conducted a sensitivity analysis using a set of medicines selected by an oncologist as first line drugs used for the management of each of seven cancers. (NM skin cancer was excluded, as no select medicines are required for its treatment based on standard treatment guidelines.) Medicines used as supportive care, such as analgesics, were excluded. For each cancer, four models were run: all medicines unadjusted, all medicines adjusted, oncologist selected medicines unadjusted, and oncologist selected medicines adjusted.

### 2.5 Role of the funding source

The study was supported by funding from the Canadian Institutes of Health Research (CIHR), Ontario SPOR Support Unit, St Michael's Hospital Foundation, and Canada Research Chairs Program. The funding source played no role in study design; in the collection, analysis, and interpretation of data; in the writing of the report; and in the decision to submit the paper for publication.

## 3 RESULTS

We collected health outcome score data for 131 of the 137 countries with NEMLs for each of the eight cancer causes (see Appendix A; Table A1). Cook Islands, Nauru, Niue, Palau, Tuvalu, and Saint Kitts and Nevis were excluded from the analysis as no cancer health outcome score data was available for them. The following countries were excluded from the analysis due to missing covariate data: Cook Islands, Cuba, Democratic People's Republic of Korea, Djibouti, Eritrea, Niue, Somalia, Syrian Arab Republic, and Venezuela. A total of 124 countries with HAQ scores and GDP data were included in this analysis.

National essential medicines lists included a median of 81 (IQR: 55.75 (56.25–112), range: 1–177) cancer treatments. The most commonly listed medicines were morphine (included on 95% of lists), methotrexate (92%) and medroxyprogesterone (87%). The number of medicines recorded for each cancer was: 10 medicines for NM skin cancer, 6 for uterine cancer, 37 for breast cancer, 36 for Hodgkin's lymphoma, 18 for colon cancer, 71 for leukemia, 12 for cervical cancer, and 13 for testicular cancer. The number of medicines selected by the oncologist for the sensitivity analysis were: 5 for uterine cancer, 3 for testicular cancer, 4 for colon cancer, 2 for cervical cancer, 7 for Hodgkin's lymphoma, 24 for leukemia, and 10 for breast cancer. The median medicine coverage scores and IQR for each cancer were: NM skin cancer 4 (IQR 3 (3–6)), uterine cancer 4 (IQR 2 (3–5)), breast cancer 12 (IQR 11 (8–19)), Hodgkin's lymphoma 17 (IQR 9 (12–21)), colon cancer 6 (IQR 5 (4–9)), leukemia 22 (IQR 17 (14–31), cervical cancer 5 (IQR 3 (4–7), and testicular cancer 8 (IQR 7 (3–10)).

**FIGURE 1:**
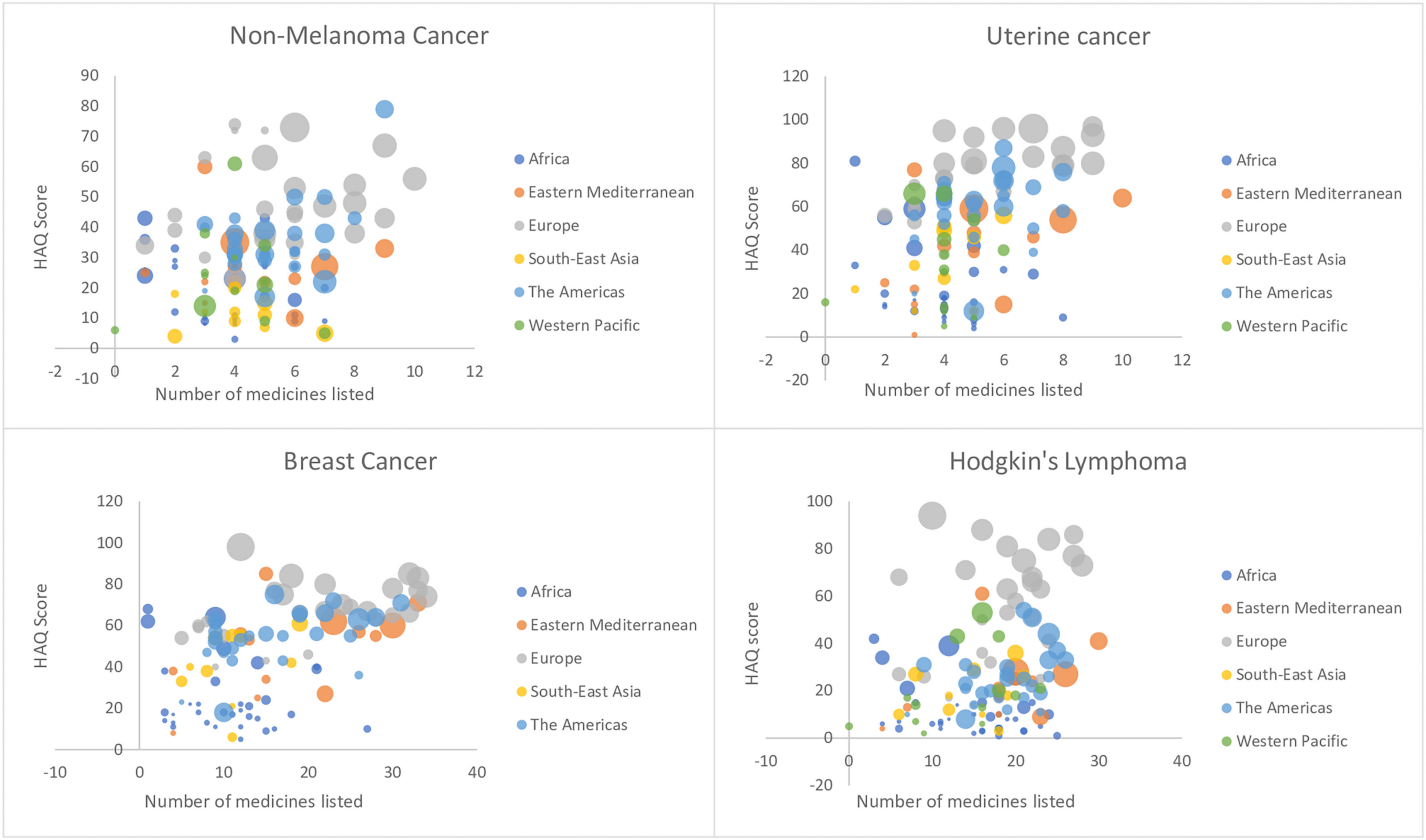
Relationship between number of medicines listed and HAQ scores by regions, with the size of the bubbles representing GDP.

**FIGURE 2:**
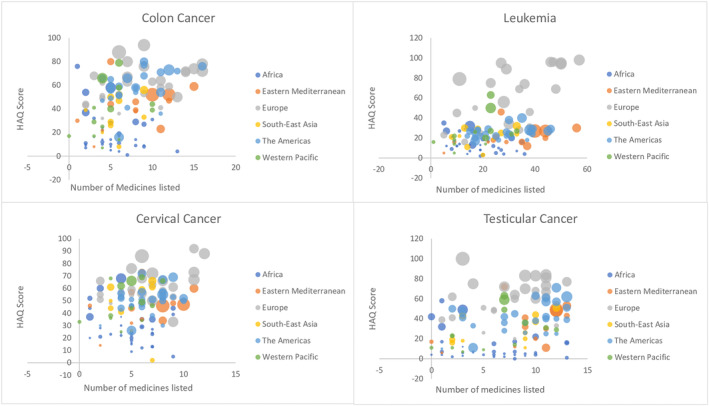
Relationship between number of medicines listed and HAQ scores by regions, with the size of the bubbles representing GDP.

The adjusted relationship between variables by regions, with the size of the bubbles representing GDP for all eight cancers is shown in Figures 1 and 2. Briefly, there were associations between medicine listing and outcomes for three of the eight examined cancers (Hodgkin's lymphoma (*p* = 0.0015), testicular (*p* = 0.0002), and cervical (*p* = 0.0004)) and the multiple linear regression model indicated that there was no association between the number of medicines listed and the health outcomes scores when GDP was taken into consideration. For each of the eight cancer causes, the relationship between listing medicines and cancer outcomes are detailed below.

### 3.1 Non‐melanoma skin cancer

The unadjusted linear regression model showed that the number of medicines listed on NEMLs account for 9.64% of the differences in cancer health outcome scores between countries (*R* ^2^ = 0.096). Adjusting the regression for GDP showed that 20% of the difference in HAQ scores for NM skin cancer (*R* = 0.20) was explained by listing medicines for NM skin cancer and taking the covariate into consideration. In the GDP unadjusted model (*p* = 0.000208), there was an association between listing the medicines and the HAQ scores. When GDP was taken into consideration with the adjusted model, this association was lost (*p* = 0.224) (see Figure 1).

### 3.2 Uterine cancer

For uterine cancer, all four models showed no association between listing medicines and HAQ scores. An unadjusted regression model showed listing medicines for uterine cancer explained approximately 20% of the differences in HAQ scores (*R* ^2^ = 0.1974). For the medicines selected by the oncologist, the model showed listing these medicines accounted for 14% difference in countries' HAQ scores (*R* ^2^ = 0.14). For the GDP adjusted model, 64% of differences in the HAQ score for uterine cancer (*R* ^2^ = 0.64) was explained by listing medicines for uterine cancer along with the GDP. For the medicines selected by the specialist, listing these medicines for uterine cancer explained the same difference of 65% (*R* ^2^ = 0.65) (see Figure 1).

### 3.3 Breast cancer

Like uterine cancer, all four models showed there was no association between listing breast cancer medicines and HAQ scores. The unadjusted model showed listing medicines for breast cancer explained approximately 24% of the differences in HAQ scores (*R* ^2^ = 0.2436). For the medicines selected by the specialist, the model showed listing these medicines accounted for 19% difference in countries' HAQ scores (*R* ^2^ = 0.19). For the GDP adjusted model, 64% of differences in the HAQ score for uterine cancer (*R* ^2^ = 0.64) were explained by listing medicines for breast cancer along with the GDP. For the medicines selected by the specialist, listing these medicines for breast cancer showed the same extent of difference in HAQ scores for uterine cancer (*R* ^2^ = 0.64) (see Figure 1).

### 3.4 Hodgkin's lymphoma

For Hodgkin's lymphoma, the unadjusted model indicated that there was an association between listing medicines for HL and HAQ scores (*p* = 0.001). Listing one of these medicines was associated with a 1.002 increase in HAQ scores. This relationship was not significant with the medicines selected by the specialist (*p* = 0.114). With adjustment for GDP, neither model showed any association between listing medicines for HL and HAQ scores. For the adjusted model, 67% of differences in the HAQ score for Hodgkin's lymphoma (*R* ^2^ = 0.66) were explained by listing medicines for HL along with the GDP. This was also the same for the medicines specially selected (*R* ^2^ = 0.66) (see Figure 1).

### 3.5 Colon cancer

The results of the unadjusted analysis for colon cancer indicated that listing all the medicines for colon cancer had no association with HAQ scores (*p* = 9.219). With listing the medicines selected by the oncologist, this relationship was present (*p* = 0.0003). Listing each medicine selected by the oncologist was associated with a 5.3 increase in HAQ scores. 17% of the difference in HAQ scores is explained by listing the total number of medicines (*R* ^2^ = 0.17) while only 10% of this difference is explained by countries' listing the specially selected medicines (*R* ^2^ = 0.095). The results of the adjusted analysis for GDP showed no association between listing medicines for colon cancer and HAQ scores. For these models, 59% of the difference in HAQ scores is explained by listing the total number of medicines and GDP (*R* ^2^ = 0.59). This was also the same association with the oncologist selected medicines (*R* ^2^ = 0.59) (see Figure 2).

### 3.6 Leukemia

For leukemia, the unadjusted analysis indicated that listing all the medicines for Leukemia had no association with HAQ scores (*p* = 0.545). With the medicines selected by the oncologist, this relationship was present (*p* = 0.0001). Listing each medicine selected by the specialist was associated with a 1.4 increase in HAQ scores. 16% of the difference in HAQ scores is explained by listing the total number of medicines (*R* ^2^ = 0.16) while 11% of this difference is explained by countries' listing the specially selected medicines (*R* ^2^ = 0.11). The results of the adjusted analysis for GDP showed no association between listing medicines for colon cancer and HAQ scores. For these models, 52% of the difference in HAQ scores is explained by listing the total number of medicines and GDP (*R* ^2^ = 0.5181). This was also similar to the association with the specialist selected medicines (*R* ^2^ = 0.5139) (see Figure 2).

### 3.7 Cervical cancer

For cervical cancer, the unadjusted analysis showed that there was a relationship between listing medicines and HAQ scores. For the total number of medicines, listing one of these medicines was associated with a 2.3 increase in HAQ scores (*p* = 0.0004) while listing any of the specialist selected medicines was associated with a 5.3 increase in HAQ scores (*p* = 0.009). This association was not observed when the models were adjusted for GDP (see Figure 2).

### 3.8 Testicular cancer

The unadjusted analysis showed that there was a relationship between listing medicines for testicular cancer and HAQ scores. For the total number of medicines, listing one of these medicines was associated with a 2.0 increase in HAQ scores (*p* = 0.0002) while listing any of the specialist selected medicines was associated with a 4.4 increase in HAQ scores (*p* = 0.014). This association was lost when the models were adjusted for GDP (see Figure 2).

## 4 DISCUSSION

Listing essential medicines for Hodgkin's lymphoma (*p* = 0.0015), testicular cancer (*p* = 0.0002), and cervical cancer (*p* = 0.0004) was associated with better disease‐specific avoidable mortality but this relationship was not present for five other examined cancers (NM skin cancer, uterine cancer, breast cancer, colon cancer, and leukemia), or for any of the eight examined cancers when models were adjusted for GDP. These results could be explained by the listed medicines not being available, by aspects of cancer care outside of medicine access, or by limits of the benefits of cancer treatments.

Cancer treatments included in NEMLs are not always available^21^; therefore, merely listing these medicines will not necessarily have an effect on amenable mortality. Shortages of anticancer medicines listed on NEMLs have been reported in a number of countries.^22^ These shortages are not limited to low‐ and middle‐income countries (LMICs) and sometimes affect high‐income countries.^23^ In high‐income countries, these shortages have been linked to a lack of financial gains for generic drug manufacturers of inexpensive anticancer medicines, while in LMICs, shortages are caused by poor infrastructures to purchase these medicines.^22^ A study in Botswana reported shortages in 50% of drugs listed on the NEML, including cancer medicines.^24^ Screening, diagnostics, surgery, and radiation are other factors that affect cancer outcomes.

Several studies have attributed higher survival rates of cancer patients in high‐income countries to greater investments in screening and diagnostic services when compared to low‐income countries.^25^, ^26^, ^27^, ^28^, ^29^ Cancer treatment efficacy depends on early and accurate diagnosis.^30^ Some LMICs lack the ability to determine whether breast cancers are estrogen dependent.^9^, ^29^ With cervical cancer, treatment outcomes are better with the concomitant use of cisplatin and radiotherapy.^31^ Adjuvant treatment modalities may also explain variation in outcomes. The use of cisplatin alone in settings with no radiotherapy centers is likely to have no curative effect on patients with stage 2B cervical cancer.^30^ Cancer mortality is related to the availability of radiation.^32^ The lack of effective screening, diagnosis and adjuvant treatments could together explain variation in outcomes.^25^, ^29^, ^33^, ^34^

There are concerns that some newer cancer treatments provide little benefit in general and, together with our findings, this may indicate that recently developed cancer treatments will not affect global mortality regardless of the clinical effects of these newer treatments. Our findings indicate that there is only a weak link between listing established cancer treatments and avoidable cancer mortality even for older proven medicines, so it would be surprising if listing newer treatments resulted in large benefits. Studies of some newer cancer treatments have a high risk of bias based on study design, conduct, or analysis.^35^ Some medicines gain approval by the European Medicines Agency and Food and Drug Administration without any randomized controlled trials demonstrating evidence of the medicine's efficacy and some recently approved cancer medicines gained market access with “conditional marketing authorizations” to treat conditions with unmet medical needs.^36^ Any expected benefit of adding newer cancer treatments to NEMLs should be balanced against the financial costs. In 2010 the cost of cancer care was >$124 billion in the United States and is expected to rise by 2020.^37^ By 2040, 65% of cancer incidences will occur in LMICs sand 71% of cancer deaths will occur in these regions.^33^ Our findings underline the need to attend to all aspects of cancer care including screening and diagnosis.

### 4.1 Strengths and limitations

We included a large number of countries and tracked disease‐specific avoidable mortality. The main findings did not depend on our classification of medicines. Accuracy of the subcomponents of the HAQ score depends on deaths being classified properly. Medicines listed on NEMLs may be unaffordable and therefore not readily available for use especially in LMICs.^22^, ^23^, ^24^ Screening, diagnostics, surgery, and radiation also influence cancer outcomes and were not the focus of this study. Age standardized mortality rates were used from breast cancer broadly and this may be a limitation. Finally, the most recent NEMLs were obtained for the year of study from the WHO repository, but newer NEMLs may be posted elsewhere.^15^

## 5 CONCLUSION

There was a weak association between listing medicines and avoidable cancer mortality. GDP seems to be a better predictor of avoidable mortality perhaps because cancer depends on non‐medicinal interventions such as screening, early detection, accurate diagnosis, access to surgery, targeted therapy, chemotherapy and radiotherapy, and actual availability of medicines listed. Further studies are required to explore the association of these interventions and health outcome scores.

## AUTHOR CONTRIBUTIONS

**Oghenefejiro (Theresa) Ikpeni:** Conceptualization (equal); data curation (equal); formal analysis (equal); validation (equal); writing – original draft (equal); writing – review and editing (equal). **Darshanand Maraj:** Conceptualization (equal); data curation (equal); formal analysis (equal); validation (equal); writing – review and editing (equal). **Hannah Woods:** Data curation (equal); formal analysis (equal); writing – review and editing (equal). **Aine Workentin:** Data curation (equal); formal analysis (equal); writing – review and editing (equal). **Christopher M. Booth:** Conceptualization (equal); supervision (equal); writing – review and editing (equal). **Nav Persaud:** Conceptualization (equal); formal analysis (equal); funding acquisition (equal); methodology (equal); supervision (equal); validation (equal); writing – original draft (equal); writing – review and editing (equal).

## FUNDING INFORMATION

The study was supported by funding from the Canadian Institutes of Health Research (CIHR), Ontario SPOR Support Unit, St Michael's Hospital Foundation, and Canada Research Chairs Program. The funders were not involved in the study design; in the collection, analysis and interpretation of the data; in the writing of the report; and in the decision to submit the paper for publication.

## CONFLICT OF INTEREST STATEMENT

NP reports grants from the CIHR, the Ontario SPOR Support Unit, the Canada Research Chairs Program and Physicians Services Incorporated during the conduct of the study. OI, DM, HW, AW and CB declare no competing interests.

## Data Availability

The data that support the findings of this study are available in the Global Essential Medicines database at https://global.essentialmeds.org/dashboard/countries.

## APPENDIX A

**TABLE A1:** Medicine coverage score^a^ for each cancer per Country.

Country	Health expenditure (PPP, 2016; $Int'l)	Total country population (2016)	NEML year	Medicine coverage score: NM Skin	Medicine coverage score: uterine	Medicine coverage score: Breast	Medicine coverage score: Hodgkin's	Medicine coverage score: colon	Medicine coverage score: leukemia	Medicine coverage score: cervical	Medicine coverage score: testicular
Afghanistan	234	34,636,207	2014	3	3	4	4	3	5	2	1
Albania	592	2,876,101	2011	2	3	10	9	2	13	2	1
Algeria	768	40,339,329	2016	1	3	10	7	2	16	1	1
Angola	193	29,154,746	2008	5	5	10	14	6	17	5	4
Antigua and Barbuda	1020	90,564	2007	1	1	1	3	1	5	1	1
Argentina	1832	43,590,368	2011	5	5	22	25	8	39	6	11
Armenia	1065	2,865,835	2010	4	3	8	16	4	18	4	6
Bahrain	2176	1,409,661	2015	4	5	23	20	10	40	8	12
Bangladesh	95	159,784,568	2008	2	1	6	12	2	11	3	3
Barbados	1066	278,649	2011	4	6	23	21	9	30	6	10
Belarus	1054	9,469,379	2012	6	5	19	20	10	34	5	8
Belize	441	367,313	2008	7	7	11	21	7	18	8	7
Bhutan	353	749,761	2016	4	4	8	12	5	14	4	2
Bolivia (Plurinational State of)	512	11,263,015	2011	6	5	17	19	8	26	7	11
Bosnia and Herzegovina	1209	3,480,986	2009	2	2	5	6	2	5	2	2
Botswana	960	2,352,416	2012	3	4	9	17	4	23	4	6
Brazil	1307	206,859,578	2014	3	4	9	9	4	15	4	2
Bulgaria	1491	7,127,822	2011	1	4	16	6	3	10	2	2
Burkina Faso	113	19,275,498	2014	5	5	12	16	6	19	5	6
Burundi	62	10,903,327	2012	3	4	4	12	3	10	4	4
Cambodia	225	15,624,584	2003	0	0	0	0	0	1	0	0
Cameroon	127	23,711,630	2010	5	5	18	21	9	27	7	10
Cape Verde (Cabo Verde)	306	558,394	2009	5	7	21	24	9	35	9	13
Central African Republic	46	4,904,177	2009	3	3	6	13	4	13	4	3
Chad	85	14,592,585	2007	4	4	8	11	4	11	5	5
Chile	1997	18,083,879	2005	5	6	16	22	7	35	9	12
China	659	1,387,790,000	2012	5	4	11	13	6	23	4	7
Colombia	1040	47,625,955	2011	7	7	19	21	9	29	8	8
Congo	107	5,186,824	2013	5	4	15	17	6	20	6	8
Cook Islands			2007	1	2	3	7	1	7	1	0
Costa Rica	1402	4,945,205	2014	6	6	19	19	11	30	8	13
Côte d'Ivoire (Ivory Coast)	158	24,213,622	2014	7	8	27	25	13	36	9	13
Croatia	1692	4,174,349	2010	8	9	33	27	14	47	11	13
Cuba	2494	11,342,012	2012	5	5	22	24	8	39	7	12
Czechia (Czech Republic)	2568	10,566,332	2012	9	8	32	24	16	50	11	10
Democratic Peoples Republic of Korea (North Korea)	25,389,611	2012	4	3	8	8	4	9	4	3	
Democratic Republic of Congo	42	81,430,977	2010	5	4	15	17	6	20	6	8
Djibouti	129	1,023,261	2007	2	2	2	8	2	8	2	1
Dominica	612	70,075	2007	4	4	9	14	5	17	4	3
Dominican Republic	939	10,527,592	2015	4	4	15	19	6	25	7	11
Ecuador	803	16,439,585	2013	8	8	25	22	13	39	10	13
Egypt	600	99,784,030	2012	4	4	12	18	5	25	5	9
El Salvador	619	6,250,510	2009	4	3	13	18	5	29	5	9
Eritrea	60	3,365,287	2010	3	3	6	15	4	20	3	3
Estonia	2030	1,315,790	2012	7	6	17	16	7	29	5	4
Ethiopia	68	105,293,228	2014	7	5	16	23	9	33	7	8
Fiji	414	918,371	2015	5	5	13	18	6	23	6	7
Gambia (The Gambia)	76	2,317,206	2001	2	2	3	4	2	6	2	1
Georgia	955	3,727,505	2007	3	3	7	17	4	19	4	6
Ghana	169	29,554,303	2010	5	4	13	18	6	24	5	6
Grenada	667	119,966	2007	4	4	9	14	5	17	4	3
Guinea	112	11,930,985	2012	2	3	4	10	2	10	3	2
Guyana	454	759,087	2010	5	4	10	14	5	14	5	4
Haiti	148	10,713,849	2012	3	3	5	7	4	9	2	1
Honduras	396	9,460,798	2009	6	7	26	23	11	37	8	12
India	205	1,338,636,340	2015	4	3	18	19	9	33	5	9
Indonesia	317	261,850,182	2011	5	5	12	21	6	27	7	10
Iran (Islamic Republic of)	1201	83,306,231	2014	9	10	33	30	15	56	11	13
Iraq	331	38,697,943	2010	6	6	22	23	11	37	8	11
Jamaica	524	2,802,695	2012	6	6	17	24	8	31	9	12
Jordan	676	9,964,656	2011	6	7	28	22	12	44	9	13
Kenya	187	47,894,670	2016	6	6	21	22	10	31	9	13
Kiribati	206	118,513	2009	4	4	7	9	4	9	4	3
Kyrgyzstan	300	6,079,500	2009	5	4	15	12	6	19	3	5
Latvia	1631	1,959,537	2012	5	5	27	14	7	23	6	8
Lebanon	1225	6,258,619	2014	3	3	15	16	5	27	6	7
Lesotho	263	2,143,872	2005	2	1	3	8	3	11	1	1
Liberia	135	4,706,097	2011	1	2	4	6	2	8	1	0
Lithuania	2038	2,868,231	2012	5	4	24	19	7	27	7	9
Madagascar	84	25,501,941	2008	3	3	7	15	4	15	4	4
Malawi	113	17,405,624	2015	4	4	12	19	4	19	6	9
Malaysia	942	31,526,418	2014	3	3	11	16	4	23	5	7
Maldives	1790	454,252	2011	5	4	9	8	6	13	3	3
Mali	83	18,700,106	2012	4	5	14	18	8	22	7	9
Malta	3599	455,356	2008	5	5	18	21	9	28	7	9
Marshall Islands	532	48,329	2007	3	4	5	7	3	9	3	2
Mauritania	146	4,051,890	2008	2	2	3	6	2	8	2	1
Mexico	1073	121,519,221	2011	9	8	31	26	16	46	10	12
Mongolia	475	3,029,555	2009	4	4	10	18	5	21	6	9
Montenegro	1559	622,303	2011	5	4	25	22	11	38	7	11
Morocco	378	35,107,264	2012	5	5	13	18	8	24	7	11
Mozambique	98	27,696,493	2016	4	4	9	11	5	14	5	3
Myanmar	206	51,892,349	2010	5	5	11	18	6	20	7	9
Namibia	972	2,323,352	2016	6	5	14	21	8	31	7	10
Nauru	1462	11,437	2010	3	4	5	7	3	7	3	2
Nepal	162	27,861,186	2011	3	3	11	16	4	24	5	9
Nicaragua	458	6,389,235	2011	3	3	8	15	4	22	5	9
Nigeria	191	188,666,931	2010	5	5	9	16	5	16	6	6
Niue			2006	2	3	3	7	2	7	2	1
Oman	1260	4,398,070	2009	7	8	30	26	12	44	10	12
Pakistan	128	213,524,840	2016	4	3	15	18	8	29	6	10
Palau	2189	17,816	2006	3	4	3	9	3	8	3	2
Papua New Guinea	97	8,899,169	2012	4	4	9	16	4	17	5	8
Paraguay	804	6,266,615	2009	4	4	12	16	6	21	6	10
Peru	607	31,132,779	2012	6	5	21	23	9	38	8	11
Philippines	305	104,875,266	2008	7	6	22	23	10	33	8	11
Poland	1851	37,970,087	2017	6	7	30	22	13	46	9	11
Portugal	2965	10,325,452	2011	8	8	22	19	9	30	8	7
Republic of Moldova	631	2,803,186	2011	6	5	20	23	11	30	7	12
Romania	1211	19,702,267	2012	9	8	32	23	14	48	9	11
Russian Federation	1289	144,342,397	2014	4	4	22	22	11	36	6	10
Rwanda	131	11,930,899	2010	4	4	7	11	5	15	4	3
Saint Kitts and Nevis	1404	47,788	2007	4	4	9	15	5	18	4	3
Saint Lucia	702	176,413	2007	4	4	9	15	5	18	4	3
Saint Vincent and the Grenadines	519	105,963	2010	5	5	11	17	6	19	6	7
Senegal	131	14,751,356	2013	6	5	15	18	7	20	7	8
Serbia	1338	7,058,322	2010	6	6	30	19	12	34	8	10
Seychelles	1316	94,677	2010	4	3	9	12	5	15	4	3
Slovakia	2111	5,430,798	2012	8	9	34	27	15	57	11	11
Slovenia	2873	2,065,042	2017	10	9	33	28	16	50	12	11
Solomon Islands	115	628,102	2017	5	5	10	16	6	21	6	5
Somalia		14,292,847	2006	0	0	0	2	0	3	0	0
South Africa	1111	56,422,274	2014	1	2	1	4	2	6	2	0
Sri Lanka	476	21,203,000	2013	2	4	11	15	5	18	6	10
Sudan	305	39,377,169	2014	1	2	4	7	1	9	1	0
Suriname	903	581,453	2014	4	4	9	19	5	22	6	7
Sweden	5513	9,923,085	2016	6	7	12	10	6	11	6	3
Syrian Arab Republic		18,964,252	2008	6	8	32	26	11	41	9	12
Tajikistan	217	8,725,318	2009	4	4	9	15	4	20	4	8
Thailand	624	70,607,037	2013	7	6	19	20	9	33	7	12
The former Yugoslav Republic of Macedonia (North Macedonia)	962	2,072,490	2008	6	6	28	24	9	38	9	12
Timor‐Leste	216	1,224,562	2015	4	4	5	6	5	8	3	2
Togo	105	7,661,354	2012	4	4	12	15	5	19	5	7
Tonga	313	105,707	2007	3	4	5	8	3	9	3	2
Trinidad and Tobago	1803	1,469,330	2010	7	6	26	24	12	38	8	13
Tunisia	685	11,685,667	2012	4	5	26	20	8	35	5	9
Tuvalu	627	10,852	2010	4	4	7	7	4	7	3	2
Uganda	108	38,748,299	2012	4	4	12	20	6	26	7	7
Ukraine	803	45,004,673	2009	3	3	7	16	4	17	5	5
United Republic of Tanzania	95	54,401,802	2013	6	4	13	16	8	19	5	6
Uruguay	1948	3,413,766	2011	7	6	28	24	11	45	8	11
Vanuatu	86	283,218	2006	3	4	6	8	3	9	3	2
Venezuela (Bolivarian Republic of)	567	30,741,464	2004	5	5	13	21	6	25	7	10
Viet Nam	372	93,126,529	2008	5	4	19	20	10	31	7	12
Yemen		29,274,002	2009	3	3	14	18	4	22	5	8
Zambia	152	16,767,761	2013	3	3	10	18	4	25	5	8
Zimbabwe	215	14,452,704	2011	4	4	11	21	5	28	6	10

^a^ Coverage score was calculated by coding the medications associated with each cancer into the GEM database, and then the medications overlapping on these lists and each country's NEML were identified and totaled, resulting in a coverage score per cancer cause.

Cancer medicines selected by oncologist for sensitivity analysis

**Table:**

Uterine cancer
Megestrol
Medroxyprogesterone
Gestonorone
Carboplatin
Paclitaxel
Testicular cancer
Etoposide bleomycin
Cisplatin
Colon cancer
Fluorouracil
Capecitabine
Oxaliplatin
Irinotecan
Cervical cancer
Cisplatin
Carboplatin
Hodgkin
Dacarbazine
Vincristine
Doxorubicin
Bleomycin
Cisplatin
Gemcitabione
Dexamethasone
Leukemia
Vitamin A (Retinol)
Tretinoin
Isotretinoin
Any selected glucocorticoid (dexamethasone, methylprednisolone, prednisone, hydrocortisone, cortisone)
Cyclophosphamide
Chlorambucil
Melphalan
Mercaptopurine
Cladribine
Fludarabine
Cytarabine
Vinblastine
Daunorubicin
Rituximab
Imatinib
Dasatinib
Nilotinib
Asparaginase
Pegaspargase
Arsenic
Methotrexate
MESNA
Calcium folinate (folinic acid, levoleucovorin, leucovorin)
Rasburicase
Breast cancer
Cyclophosphamide
Paclitaxel
Docetaxel
Doxorubicin
Epirubicin
Trastuzumab
Tamoxifen
Anastrozole
Letrozole
Exemestane
